# Dietary Supplementation with a Superoxide Dismutase-Melon Concentrate Reduces Stress, Physical and Mental Fatigue in Healthy People: A Randomised, Double-Blind, Placebo-Controlled Trial

**DOI:** 10.3390/nu6062348

**Published:** 2014-06-19

**Authors:** Julie Carillon, Claire Notin, Karine Schmitt, Guy Simoneau, Dominique Lacan

**Affiliations:** 1 Bionov Company, 939 rue de la Croix Verte, Montpellier 34090, France; E-Mails: jc.rech@bionov.fr (J.C.); karine.schmitt@bionov.fr (K.S.); 2 Seppic Company, 22 Terasse Bellini, Puteaux 92806, France; E-Mail: claire.notin@airliquide.com; 3 Therapeutic Research Unit, Department of Internal Medicine, Lariboisière Hospital, 2 rue Ambroise-Paré, Paris 75010, France; E-Mail: guy.simoneau@lrb.aphp.fr

**Keywords:** psychological stress, physical and mental fatigue, oxidative stress, melon, superoxide dismutase, psychometric scales

## Abstract

Background: We aimed to investigate effects of superoxide dismutase (SOD)-melon concentrate supplementation on psychological stress, physical and mental fatigue in healthy people. Methods: A randomized, double-blind, placebo-controlled trial was performed on 61 people divided in two groups: active supplement (*n* = 32) and placebo (*n* = 29) for 12 weeks. Volunteers were given one small hard capsule per day. One capsule contained 10 mg of SOD-melon concentrate (140 U of SOD) and starch for the active supplement and starch only for the placebo. Stress and fatigue were evaluated using four psychometric scales: PSS-14; SF-36; Stroop tests and Prevost scale. Results: The supplementation with SOD-melon concentrate significantly decreased perceived stress, compared to placebo. Moreover, quality of life was improved and physical and mental fatigue were reduced with SOD-melon concentrate supplementation. Conclusion: SOD-melon concentrate supplementation appears to be an effective and natural way to reduce stress and fatigue. Trial registration: trial approved by the ethical committee of Poitiers (France), and the ClinicalTrials.gov Identifier is NCT01767922.

## 1. Introduction

Modern life induces situations and pressure that cause stress. Each individual has his/her own level of stress tolerance. What may be overwhelming for one person is someone else’s challenge. When stress acts as a positive motivating force it is termed eustress. When it acts as a negative force it is termed distress. Stress only becomes a problem when it is chronic or severe. Indeed, long-term exposure to stress can lead to complications, such as anxiety, depression, social isolation, intense mood swings, physical and mental fatigue, but also more severe problems, such as cardiovascular diseases or immune system suppression. It is thus essential to prevent phsychological stress to avoid all related disorders, which can be severe.

Psychological stress and fatigue may lead to oxidative stress.

The link between individual stress and intracellular oxidative stress is now well-established. Indeed, Casado *et al*. have demonstrated in a clinical trial on nurses in an intensive care unit, a clear relationship between oxidative stress markers, in particular an increase of reactive oxygen species (ROS) production and malondialdehyde (MDA) levels, and occupational stress [1,2]. In the same way, Lucca *et al*. showed, on a rat model of depression, that individual stress induces oxidative stress (increase of lipid peroxidation) and an imbalance between antioxidant enzymes activities (superoxide dismutase (SOD) and catalase) that contribute to stress-related diseases like depression [3]. Finally, Maes *et al*. have shown that major depression is accompanied by a decrease in zinc, coenzyme Q10, vitamin E and antioxidant enzymes, and by an induction of oxidative and nitrosative pathways [4,5].

Oxidative stress is also involved in fatigue. Shichiri *et al*. have demonstrated an increase in oxidative stress markers in plasma of tired patients, who worked overnight [6]. Oxidative stress levels are also raised in chronic fatigue syndrome [7]. Indeed, Morris and Maes [8] have reviewed that chronic fatigue syndrome is accompanied by lowered zinc, and reduced glutathione and coenzyme Q10.

In summary, oxidative stress (increase in ROS and decrease of antioxidant defenses) is involved in psychological stress and related disorders.

In this context, it could be interesting to consider supplementation based on antioxidants as a possible strategy for improving stress and related disorders. An improvement in memory has been reported by Tagliari *et al*. in stressed rats treated with vitamins C and E [9]. Gupta *et al*. have shown that curcumin attenuates chronic fatigue syndrome in murine water immersion stress model [10]. Some human clinical studies have also demonstrated beneficial effects of antioxidants on stress and fatigue. For example, Yagi *et al*. have shown that supplementation with lutein could help to reduce symptoms of visual fatigue [11]. Oral administration of pyrroloquinoline quinone reduces stress, fatigue, and improves sleep [12].

Moreover, the influence of Extramel^®^ (Bionov, Eyragues, France), a form of melon SOD, has been assessed in a first pilot study, during 28 days [13], in which beneficial effects have been shown.

The present clinical study aims to confirm the results of our earlier pilot study and know whether the daily intake of this SOD melon concentrate can reduce stress and fatigue in human patients over a longer period (84 days).

## 2. Experimental Section

### 2.1. Test Material

Extramel^®^ is a proprietary coated freeze-dried melon juice concentrate obtained by physical treatment (crushing the melon, recovery of the pulp, centrifugation, filtration, freeze-drying, coating) of a specific variety of melon (not genetically modified organism) which contains high levels of SOD and other antioxidants [14] (Table 1). Due to its high levels, we assume that SOD is the primary bioactive compound of Extramel^®^. For nutraceutical and experimental applications, Extramel^®^ is coated with palm oil in order to protect the SOD activity from digestive enzymes. In this study, it contains 14 U SOD/mg powder measured according to the method of Zhou and Prognon [15]. Volunteers were given one small hard capsule per day. One capsule contains 10 mg Extramel^®^ (140 U of SOD) and starch for the active supplement (S), and starch only for the placebo (P).

**Table 1:** Antioxidants composition of Extramel^®^.

Antioxidants	Level (per g)
Superoxide dismutase	14,000 U
Catalase	1550 U
Glutathione peroxydase	155 U
Co-enzyme Q10	0.08 mg
Lipoic acid	0.03 mg
Glutathione	0.33 μg
Glutathione disulfide	4.78 μg
Carotenoids	0.54 μg
Vitamin A	15.5 μg
Vitamin E	0.37 μg
Vitamin C	7.78 μg
Selenium	0.004 μg
Total phenolics	0.54 mg GAE ^1^

^1^ GAE: gallic acid equivalents.

### 2.2. Methods: Psychometric Tools

The efficacy of Extramel^®^ on perceived stress and fatigue on adult subjects was investigated in a controlled clinical study *vs.* placebo, randomized and double blind during 84 days, using validated psychometric scales.

The main criterion of this study concerned the evaluation on perceived stress after 84 days of supplementation. Secondary criteria assessed the consequences of stress on: quality of life, physical fatigue, and mental fatigue.

Cohen Perceived Stress scale (PSS-14) evaluates the perceived stress over the past two weeks with 14 questions and a score from 14 to 70. It was the main criterion of the study [16].

SF-36^®^ (The Health Institute, Boston, MA, USA) Health Survey allows measurement of eight aspects of the quality of life: general physical and mental health state, physical and social functioning, physical and emotional health, pain, and vitality [17]. The SF-36 questionnaire is composed of 36 questions with a score from 0 to 36.

Stroop test and reverse Stroop test is used to assess the impact of stress on the intellectual fatigue [18]. First, the subject is placed in front of a Stroop test grid, word grid written in different colors. The subject has 40 s to write the first letter of the color of the ink in which the word is printed in the corresponding box (for example Y if the word is written in yellow). Following this test, for the reverse Stroop test, the subject is placed again in front of the Stroop test grid with words written in different colors. However, this time, the subject must write down in the corresponding box the first letter of the color written on the test irrespective of the color in which the word is printed. This test is also performed for 40 s.

Results represent the cumulative score of Stroop + reverse Stroop.

Prevost subjective fatigue scale is a questionnaire intended to measure the impact of stress on the physical fatigue of the subjects [19]. The subject has to score 1 to 7 the following impact points on his/her fatigue: global fatigue perceived level, muscle pain, sleep troubles, and stress.

The Hamilton anxiety scale, a 14 questions multiple choice self-report inventory that is one of the most widely used instruments for measuring the severity of depression [20], was used to avoid the inclusion of depressive volunteers.

### 2.3. Study Design

The clinical trial was an intervention study based on the individual evaluation scales described below. The protocol followed was randomized, double blind and placebo controlled. It was approved by the Comité de Protection des Personnes Ouest III, the ethical committee of Poitiers (France), and the ClinicalTrials.gov Identifier is NCT01767922. The study was multi-centric (two average sized provincial cities); Thierry Cantin was validated as the principal investigator and Patrick Leprince as co-investigator.

A call for volunteers was made in the regions of the investigation centers and the volunteers for the study were pre-screened by the investigators.

The inclusion criteria were to be between 30 and 65 years old, to have a BMI (Body Mass Index) ≤30, to have a stable professional activity for more than one year, to perceive stress and tiredness (assessed with psychometric scales described above), to be in full health, not taking any drugs or dietary supplements, not taking anti-stress or anti-tiredness drinks.

The exclusion criteria were to be pregnant or breast-feeding, to have a previous case of psychiatric disease, to have pathologies on going or active during the last month, or to have received medical treatment (allopathic or homeopathic) during the previous month. Patients who took a dietary supplement during the last month are excluded. Finally, patients who have a special situation (wedding, birth, scheduled hospitalization, important exam, *etc*.) during the next month, which can cause a higher level of stress during the study, are also excluded.

Acceptable volunteers were called in for a screening and baseline evaluation using the five evaluation scales previously described: PSS-14, SF-36, Prevost scale, Stroop tests, and Hamilton scale. To be included, the volunteers had to get scores within the following parameters: 5 < Hamilton scale < 20; PSS14 > 30 and presenting an impact of stress on at least one of the following scale: Prevost scale > 50 or Stroop + reverse Stroop test < 160.

Then, 61 volunteers were definitively included and participated in the clinical trial. The volunteers assigned by randomization into two groups of 32 subjects for the active supplement group (S) and 29 subjects for the placebo group (P) were given a food supplement or a placebo for 84 days. The capsules were indistinguishable and were administered in a double blind approach.

The volunteers were tested three times during a visit to the doctor. The first time was before the supplementation (D0), corresponding to the screening evaluation. A test was planned 28 days (D28) after taking the supplement and another one at the end of the trial (D84). The evaluation scales were filled out by the volunteers themselves. During every consultation, the doctor made a general clinical exam (arterial tone and cardiac frequency measure) in order to detect any disorder, and completed an event journal with remarks on the volunteers, eventual undercurrent effects, the eventual pathologies, and their associated treatment.

These volunteers provided a written informed consent.

### 2.4. Statistical Analysis

The data were expressed as Means ± SEM. Changes between D0 and D28/D84 for each group (S, P) were tested using a multiple comparison test (Dunnett’s test) for repeated measures.

The heterogeneity between the two groups at baseline (D0) was tested using a non-parametric test for unpaired data (Mann-Whitney test).

The comparison between active supplement and placebo was carried out on the differences D28–D0 and D84–D0 using Mann-Whitney test.

The software used to performed statistical analysis is Statview^®^ 5.0 (Abacus Concepts, Berkeley, CA, USA).

## 3. Results

### 3.1. Study Population

Sixty one volunteers aged between 29 and 60 years (mean 43.6) were recruited and randomized into two test groups (*n* = 32 for active supplement group and *n* = 29 for placebo group). No subjects were excluded and the gender-ratio was 68% women. There were no statistical differences between the two groups at baseline.

3.2 Perceived Stress Scale

**Figure 1:**
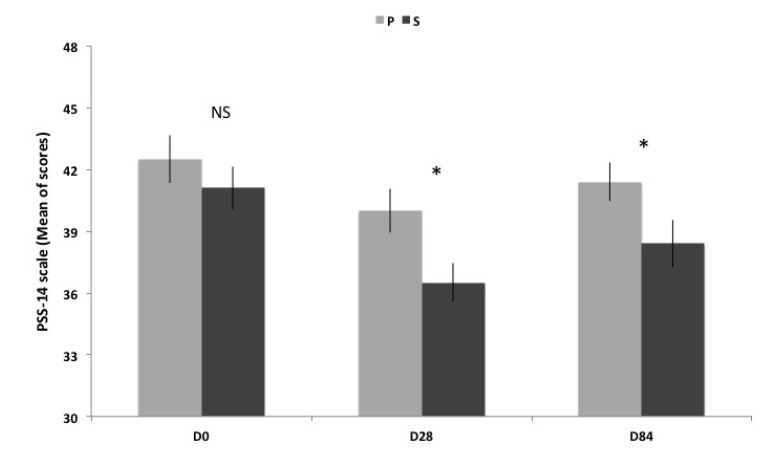
Scores of PSS-14 scale (Mean ± SEM) before, between and after supplementation. Transverse comparison between groups are not significantly different (NS) for D0, but significantly different (* *p* < 0.05) for D28 and D84.

PSS-14 was the primary criterion of the study. Results presented on Table 2 and Figure 1 show that the difference between the two groups was in favor of the S group at D28 (−8.8%) and D84 (−7.2%). Compared to P group, perceived stress was significantly less intense in the S group at D28 and D84.

**Table 2:** .Scores at D0, D28, and D84, transverse comparison between supplement group(S) and placebo group (P) and evolution between D0 and D84 for P and S groups.

Scores	Scores (Mean ± SEM)									Evolution between D0 and D84	
	D0			D28			D84			P Group	S Group
	P Group	S Group	S *vs*. P *p* Value	P Group	S Group	S *vs*. P *p* Value	P Group	S Group	S *vs*. P *p* Value	*p* Value	*p* Value
PSS-14	42.5 ± 1.2	41.1 ± 1.0	0.3629	40.0 ± 1.1	36.5 ± 0.9	<0.03	41.4 ± 0.9	38.4 ± 1.1	<0.04	>0.05	<0.01
SF-36	43.6 ± 1.6	43.1 ± 1.5	0.8057	36.3 ± 1.9	35.3 ± 1.4	>0.05	37.3 ± 2.5	34.9 ± 1.4	<0.04	<0.01	<0.01
Stroop scale	67.8 ± 2.7	73.2 ± 3.6	0.3586	80.1 ± 4.2	91.2 ± 5.0	<0.04	77.5 ± 2.9	93.6 ± 4.8	<0.001	<0.05	<0.01
Prevost scale	95.7 ± 5.2	91.8 ± 3.3	0.7233	92.3 ± 4.7	84.7 ± 3.5	>0.05	93.2 ± 4.8	84.4 ± 2.8	<0.02	>0.05	<0.01

The decrease in perceived stress occurred in both groups at D28, compared to D0. However, the placebo effect stops after D28. Indeed, compared to D0, there is a significant reduction at D28 but not at D84 in the P group, whereas a significant decrease in perceived stress occurred at D28 and D84 in the S group.

### 3.3. Quality of Life: SF 36^®^ Health Survey

**Figure 2:**
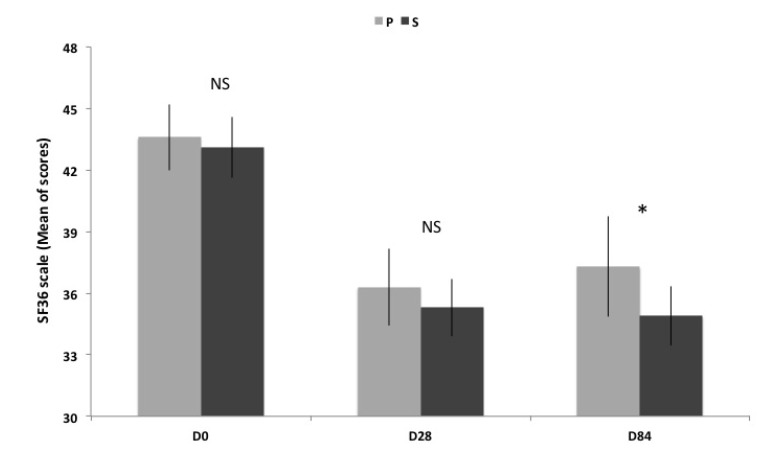
Scores of SF 36 scale (Mean ± SEM) before, between and after supplementation. Transverse comparison between groups are not significantly different (NS) for D0 and D28, but significantly different (* *p* < 0.05) for D84.

Quality of life is improved in both groups at D28 and D84 compared to D0 as reported on Table 2 and Figure 2.

However, the transverse comparison between S and P groups shows that the improvement of quality of life is significantly more important in S group than P group at D84.

### 3.4. Stroop and Reverse Stroop Test

**Figure 3:**
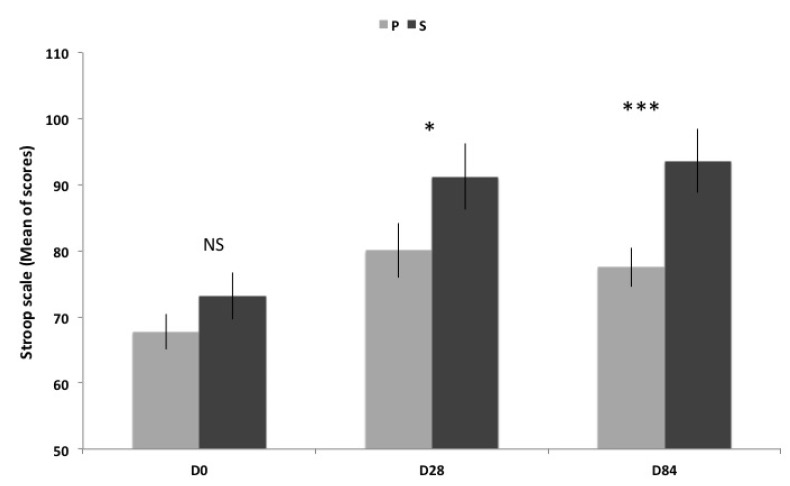
Scores of Stroop scale (Mean ± SEM) before, between and after supplementation. Transverse comparison between groups are not significantly different (NS) for D0, but significantly different (* *p* < 0.05; *** *p* < 0.001) for D28 and D84.

Mental fatigue measured with Stroop and reverse Stroop test is improved in both groups at D28 and D84 (Table 2 and Figure 3). However, regarding both groups, the improvement is better in S group compared to P group after 28 days of treatment (13.9%) and continues to increase until the end of the study (20.8%).

### 3.5. Prevost Subjective Fatigue Scale

**Figure 4:**
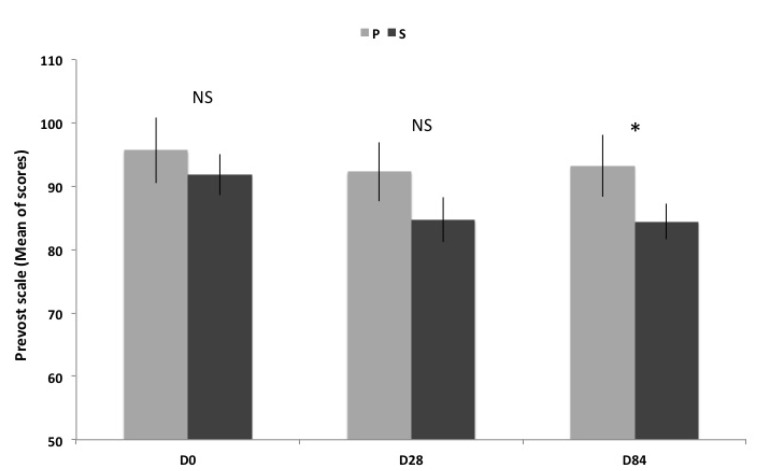
Scores of Prevost scale (Mean ± SEM) before, between and after supplementation. Transverse comparison between groups are not significantly different (NS) for D0 and D28, but significantly different (* *p <* 0.05) for D84.

As reported in Table 2 and Figure 4, the physical fatigue is not improved in P group at D28 neither at D84 and this clearly indicates that there is no placebo effect for this test. On the contrary, in S group, there is a significant reduction of perceived physical fatigue at D28 and D84. The transverse comparison of scores between S and P groups indicates that the reduction of physical fatigue is maximal at D84 and reaches 9.4% compared to P group.

## 4. Discussion

This clinical trial aimed to assess beneficial effects of a melon SOD concentrate supplementation on stress and fatigue, in healthy people. The imbalance in gender-ratio, 68% women in the present study, could be explained by the fact that perceptions of stress and responses to them may differ between men and women [21,22]. Indeed, stress-related disorders are more prominent among women than among men [23].

As reported, there is a high placebo effect that is commonly found in this type of study using subjective scales, which was also observed in the pilot study of 28 days [13]. Nevertheless, the current study shows that this placebo effect stops to evolve after 28 days of treatment.

Our results clearly show a significant improvement after Extramel^®^ supplementation compared to placebo on all the criteria investigated:

- Perceived stress (Cohen scale PSS-14) and mental fatigue (Stroop score) at D28;
- Perceived stress (Cohen scale PSS-14), physical fatigue (Prevost score), mental fatigue (Stroop score) and quality of life (SF 36) at D84.

Perceived stress, the main parameter of the study, was significantly decreased by Extramel^®^ supplementation. Quality of life (general physical and mental health state, physical and social functioning, physical and emotional health, pain, and vitality) was also improved as shown by SF-36 Health Survey scale results. Moreover, Extramel^®^ supplementation clearly reduced physical and mental fatigue. It is important to note the Stroop score improvement (27.9%) after three months of treatment. Stroop test is considered to measure selective attention, cognitive flexibility, and processing speed, and is currently used as a tool in the evaluation of executive functions.

Beneficial effects observed on quality of life, and physical and mental fatigue, may be due to the decrease of psychological stress, which is often the cause of this kind of disorder.

Houghton *et al*. have assessed the influence of another form of melon extract on fatigue [24]. However, the melon extract/gliadin supplement had no significant effect on self-perceived fatigue in women aged 50–65 years [24]. This could be explained by the difference of the melon extract formulation. Indeed, the SOD contained in Extramel^®^ is protected from digestive enzymes by a specific patented coating in order to preserve the product activity.

Psychological stress and fatigue, usually described as oxidative stress-related states, are reduced with Extramel^®^. This suggests that Extramel^®^ supplementation could act through a modulation of oxidative status, as this mechanism has already been demonstrated in other models of oxidative stress related diseases [25]. Indeed, exactly the same oral melon concentrate administration was associated with an increased in endogenous antioxidant defence in animals [26,27,28,29]. We have shown that melon SOD concentrate supplementation, could increase endogenous antioxidant enzymes (SOD, glutathione peroxidase and catalase) in the liver and adipose tissue of obese hamsters [26,27], in the liver of healthy rats [28], and in the heart of spontaneously hypertensive rat [29]. Even if experimental conditions are not exactly the same in these studies, in the last work [29], animals received 4 U SOD/day, which correspond approximately to 140 U SOD/day for humans [30]. And we have shown an induction of cardiac endogenous antioxidant defence by 40% in the supplemented rats, compared to untreated rats.

Endogenous antioxidant defence enhancement cannot be quantified in the blood. That is why it is difficult to confirm this hypothesis in humans. Oxidative stress markers, such as isoprostane levels, could be quantified in order to confirm the decrease in oxidative stress, related to endogenous antioxidant defence induction, as we have shown in the liver of supplemented obese hamsters [26].

Beneficial effects observed in this study may be due to the enhancement of endogenous antioxidant defence and the subsequent reduction of oxidative stress. However, further investigations need to be done in order to confirm this hypothesis in humans, and the precise mechanism of action remains to be investigated.

## 5. Conclusions

Extramel^®^ appears to be an effective and natural way to reduce stress and fatigue (physical and mental).
